# Detection of COVID-19 from Chest X-Ray Images Using Convolutional Neural Networks

**DOI:** 10.1177/2472630320958376

**Published:** 2020-09-18

**Authors:** Boran Sekeroglu, Ilker Ozsahin

**Affiliations:** 1 Department of Information Systems Engineering, Near East University, Nicosia/TRNC, Mersin-10, Turkey; 2 Department of Biomedical Engineering, Faculty of Engineering & DESAM Institute, Near East University, Nicosia/TRNC, Mersin-10, Turkey

**Keywords:** COVID-19, pneumonia, X-ray, convolutional neural networks, coronavirus

## Abstract

The detection of severe acute respiratory syndrome coronavirus 2 (SARS CoV-2), which is responsible for coronavirus disease 2019 (COVID-19), using chest X-ray images has life-saving importance for both patients and doctors. In addition, in countries that are unable to purchase laboratory kits for testing, this becomes even more vital. In this study, we aimed to present the use of deep learning for the high-accuracy detection of COVID-19 using chest X-ray images. Publicly available X-ray images (1583 healthy, 4292 pneumonia, and 225 confirmed COVID-19) were used in the experiments, which involved the training of deep learning and machine learning classifiers. Thirty-eight experiments were performed using convolutional neural networks, 10 experiments were performed using five machine learning models, and 14 experiments were performed using the state-of-the-art pre-trained networks for transfer learning. Images and statistical data were considered separately in the experiments to evaluate the performances of models, and eightfold cross-validation was used. A mean sensitivity of 93.84%, mean specificity of 99.18%, mean accuracy of 98.50%, and mean receiver operating characteristics–area under the curve scores of 96.51% are achieved. A convolutional neural network without pre-processing and with minimized layers is capable of detecting COVID-19 in a limited number of, and in imbalanced, chest X-ray images.

## Introduction

At the end of 2019, humankind was faced with an epidemic—severe acute respiratory syndrome coronavirus 2 (SARS CoV-2)–related pneumonia, referred to as coronavirus disease 2019 (COVID-19)—that people did not expect to encounter in the current era of technology. While the COVID-19 outbreak started in Wuhan, China, the significant spread of the epidemic around the world has meant that the amount of equipment available to doctors fighting the disease is insufficient. At the time of writing (September 8, 2020), there have been more than 27,000,000 confirmed cases and more than 875,000 confirmed deaths worldwide.^1^ Considering the time required for diagnosis and the financial costs of the laboratory kits used for diagnosis, artificial intelligence (AI) and deep learning research and applications have been initiated to support doctors who aim to treat patients and fight the illness.^2^

Although rapid point-of-care COVID-19 tests are expected to be used in clinical settings at some point, for now, turnaround times for COVID-19 test results range from 3 to more than 48 hours, and probably not all countries will have access to those test kits that give results rapidly. According to a recently published multinational consensus statement by the Fleischner Society, one of the main recommendations is to use chest radiography for patients with COVID-19 in a resource-constrained environment when access to computed tomography (CT) is limited.^3^ The financial costs of the laboratory kits used for diagnosis, especially for developing and underdeveloped countries, are a significant issue when fighting the illness. Using X-ray images for the automated detection of COVID-19 might be helpful in particular for countries and hospitals that are unable to purchase a laboratory kit for tests or that do not have a CT scanner. This is significant because, currently, no effective treatment option has been found, and therefore effective diagnosis is critical.

AI tools have produced stable and accurate results in the applications that use either image-based or other types of data.^2,4–6^ Apostolopoulos and Mpesiana^2^ performed one of the first studies on COVID-19 detection using X-ray images. In their study, they considered transfer learning using pre-trained networks such as VGG19, MobileNet V2, Inception, Xception, and Inception ResNet V2, which are the most frequently used. Several evaluation metrics were used to evaluate the results obtained from two different datasets. MobileNet V2 and VGG19 achieved 97.40% and 98.75% accuracy, respectively, for two-class experiments (COVID-19/Normal and COVID-19/Pneumonia), and 92.85% and 93.48% for three-class experiments (COVID-19/Pneumonia/Normal). The final conclusion was made by the authors using the obtained confusion matrices, not the accuracy results because of the imbalanced data.

Ozsahin et al.^4^ used the average pixel per node (APPN) approach, which is also considered in this study, and image pre-processing techniques to detect Alzheimer’s disease in positron emission tomography (PET) images. Dai et al.^5^ modeled vehicle interactions using long short-term memory neural networks and predicted the trajectory for the vehicles. Yilmaz et al.^6^ applied several machine learning classification models to classify student performance, using a numerical dataset whose implemented logistic regression (LR) and decision trees are considered in this study. All these and similar studies obtained high-accuracy results using AI techniques. For that reason, it has been widely used in the past two decades. AI, which aims to imitate human nature, can learn and makes decisions from data and images.

Deep learning, which takes its name from the number of its hidden layers, has gained a special place in the field of AI by providing successful results for both image-based classification applications and regression problems during the past 10 years.^7,8^ The frequent use of deep convolutional neural networks (ConvNet, or CNNs)^9^ has enabled image-based applications to reach their peak in the past 5 years. Generally, CNNs that try to simulate biological aspects of human beings on computers required pre-processing of images or data before feeding them to the network. When the ConvNet was first invented, however, it was described as a neural network that requires minimal pre-processing of images before feeding them to the network, and a system that is capable of extracting the features from images to optimize the learning performance of the neural network.^6^ The ConvNet comprises both feature extraction and classification phases in a single network. A traditional ConvNet consists of three layers: convolution, pooling, and fully connected layers. Feature extraction is performed in the convolutional layer by applying masks, which is the process of dividing images into a predefined dimension of segments and using filters to extract features from the image. Then a feature map, which is the projection of features on the 2D map, is created by applying an activation function to the values obtained by the masks. The activation function activates the most knowledgeable neurons in a nonlinear way and reduces the computational cost of the neural network. Several activation functions are available in CNNs, and the rectified linear unit (ReLU) is the most commonly used activation function; it does not activate all the neurons at the same time, and therefore provides a faster convergence when the weights find the optimal values to produce the trained response during the training. A pooling operation is performed on the produced feature map to reduce the dimensions of the images. Finally, the feature map is flattened into a vector and sent to the fully connected layer. The convergence of the neural network and the classification of the input patterns are performed in the fully connected layer, and its principles are based on error backpropagation to update the weights within this layer.

Deep ConvNets were applied in several image recognition applications with high accuracy, and this increased its reliability for future research.^10–12^ Roy et al.^10^ explored CNNs for hyperspectral image classification, and Hartenstein et al.^11^ used deep learning to determine prostate cancer positivity from CT imaging. Yoon et al.^12^ used a CNN for tumor identification in colorectal histology images. These and similar studies motivated researchers to investigate whether AI and ConvNets can be used effectively in COVID-19 research, particularly in diagnostic applications. Recently, Apostolopoulos and Mpesiana^2^ performed a study on the classification of novel COVID-19. They considered two different, publicly available chest X-ray images. The training process was performed by using ConvNet with transfer learning with pre-trained networks. They concluded that VGG19 and MobileNet-V2 outperformed other pre-trained ConvNets.

Each trained neural network gains knowledge for the particular task that is considered. While the main principle of artificial neural networks is to simulate human behavior and intelligence, the transfer learning in artificial neural networks is used to apply the stored knowledge of a particular task for another related task. Deep learning for image recognition applications is capable of learning millions of images, and several huge models were trained with different architectures.^13–17^ These pre-trained models have been publicly shared so that all researchers can make use of the stored knowledge. The state-of-the-art pre-trained publicly available networks, namely, VGG16,^13^ VGG19,^13^ ResNet50,^14^ InceptionV3,^15^ MobileNet-V2,^16^ and Densenet121,^17^ were considered in comparison.

When we consider the incidence rates of COVID-19, it is obvious that the data we can encounter in real life will be imbalanced. Therefore, it is important to evaluate which methods and ConvNet architectures can be used on imbalanced data, and, if efficient, which pre-processing methods. In this study, 1583 normal, 4292 pneumonia-infected (2790 bacterial and 1502 viral), and 225 COVID-19-infected original and pre-processed public X-ray images were considered for the diagnosis of COVID-19 using the different architectures of the most widely used image recognition ConvNet networks. The classification was performed between COVID-19 and normal images, COVID-19 and pneumonia images, and COVID-19, pneumonia, and normal images to provide high-efficiency detection of COVID-19 in chest X-ray images and to differentiate COVID-19 from both normal and pneumonia-infected images. The receiver operating characteristics (ROC) area under the curve (AUC) scores and macro-averaged F1 score of the pre-processed and original images were examined. In addition, five machine learning classifiers—support vector machines (SVMs), LR, naive Bayes (nB), decision tree (DT), and k-nearest neighbor (kNN)—were implemented with 14 statistical data attributes obtained from the images, and the image-based and statistically based results were compared. Finally, six state-of-the-art pre-trained ConvNets—VGG16, VGG19, InceptionV3, MobileNet-V2, ResNet50, and DenseNet121—were considered for the comparison.

According to their previously mentioned statement,^3^ the Fleischner Society recommends that medical practitioners use chest X-ray and CT in the management of COVID-19. In the end, the choice of imaging modality is left to the judgment of clinical teams at the point of care, accounting for the differing attributes of chest radiography and CT, local resources, and expertise. In this study, we propose the use of chest X-ray images over CT of the thorax, considering the latter’s required diagnostic time. A CT scan of the thorax takes significantly more time than a chest X-ray scan does, and this means more contact duration with suspected or confirmed COVID-19 patients.

## Materials and Methods

### Dataset

A total of 225 COVID-19 chest X-ray images were obtained from Cohen;^18^ they can be accessed from github.^19^ The average age for the COVID-19 group was 58.8±14.9 years, and it comprised 131 male patients and 64 female patients. Note that some patients’ information is missing; this is because the dataset used in this study does not have accompanying complete metadata, because this is the very first publicly available COVID-19 X-ray image collection, and it was created in a limited time. In addition, 1583 normal and 4292 pneumonia chest X-ray images were obtained from Kermany et al.^20^ All images were in different dimensions, so they were resized to 640 × 480.

### Design of Experiments

Several categorized experiments were performed to evaluate the efficiency of the ConvNet on the considered image database and to compare ConvNet with other models using the basic statistical characteristics of the images, which can provide effective information for classification. Experiments were divided into three categories: ConvNet experiments, statistical measurement experiments, and transfer learning experiments.

#### ConvNet Experiments

ConvNet experiments were performed on three subcategories: COVID-19/Normal, COVID-19/Pneumonia, and COVID-19/Pneumonia/Normal. They included the use of four different network architectures with varying numbers of convolutional and fully connected layers, and basic image pre-processing techniques to test the results using various structures and pre-processing methods.

The first structure (ConvNet#1) consisted of two convolutional layers with 64 and 16 filters, respectively, with two fully connected (dense) layers with 128 and 8 neurons. It was the lightest architecture considered in this study. The second and third ConvNet structures (ConvNet#2 and ConvNet#3) included three convolutional layers with 256, 128, 64 and 128, 64, 32 filters, respectively, and two fully connected layers were implemented with 128 and 8 neurons. ConvNet#4, which was the deepest architecture in this study, consisted of four convolutional layers (256, 128, 128, and 64 filters) and three fully connected layers (128, 64, and 8 neurons). The filter sizes were considered as 3×3 for all structures, and 0.2 dropout was used for each layer. Pooling was applied as maximum pooling, and 2×2 pooling was considered for each layer except the last convolutional layer of each structure. The pooling was applied as 1×1 in the last convolutional layer of each structure, to not minimize the features extracted by convolutional layers. **Table 1** and **Table 2** show the architectural properties of four considered ConvNets.

**Table 1.** Architectural Properties of Four Considered ConvNets.

Architecture Name	ConvNet Layer No.	Filters	Filter Size	Pooling and Size	Dropout	Activation
ConvNet#1	ConvNet Layer 1	64	3×3	Max-pooling 2×2	0.2	ReLU
	ConvNet Layer 2	16		Max-pooling 1×1		
ConvNet#2	ConvNet Layer 1	128	3×3	Max-pooling 2×2	0.2	ReLU
	ConvNet Layer 2	64				
	ConvNet Layer 3	32		Max-pooling 1×1		
ConvNet#3	ConvNet Layer 1	256	3×3	Max-pooling 2×2	0.2	ReLU
	ConvNet Layer 2	128				
	ConvNet Layer 3	64		Max-pooling 1×1		
ConvNet#4	ConvNet Layer 1	256	3×3	Max-pooling 2×2	0.2	ReLU
	ConvNet Layer 2	128				
	ConvNet Layer 3	128				
	ConvNet Layer 4	64		Max-pooling 1×1		

ConvNet: Convolutional neural network; ReLU: rectified linear unit.

**Table 2.** ConvNet Experiments and General Properties.

Experiment No.	ConvNet Architecture	Input Dimension	Pre-Processing	Dense Layer #1	Dense Layer #2	Dense Layer #3
Exp.1	ConvNet#1	160×120	Sharpening	128	8	—
Exp.2	ConvNet#2	160×120	Sharpening	128	8	—
Exp.3	ConvNet#3	160×120	Sharpening	128	8	—
Exp.4	ConvNet#4	160×120	Sharpening	128	64	8
Exp.5	ConvNet#1	30×20	Sharpening	128	8	—
Exp.6	ConvNet#2	30×20	Sharpening	128	8	—
Exp.7	ConvNet#3	30×20	Sharpening	128	8	—
Exp.8	ConvNet#1	30×20	APPN	128	8	—
Exp.9	ConvNet#2	30×20	APPN	128	8	—
Exp.10	ConvNet#3	30×20	APPN	128	8	—
Exp.11	ConvNet#1	160×120	—	128	8	—
Exp.12	ConvNet#2	160×120	—	128	8	—
Exp.13	ConvNet#3	160×120	—	128	8	—
Exp.14	ConvNet#4	160×120	—	128	64	8
Exp.15	ConvNet#1	30×20	—	128	8	—
Exp.16	ConvNet#2	30×20	—	128	8	—
Exp.17	ConvNet#3	30×20	—	128	8	—

APPN: Average pixel per node; ConvNet: convolutional neural network.

A total of 34 experiments were performed in this category, 17 each of COVID-19/Normal and COVID-19/Pneumonia, to evaluate and analyze the performance of ConvNets under different conditions to achieve an optimal classification of COVID-19 images. The COVID-19/Pneumonia/Normal experiments were performed by considering the optimal results obtained in the other two categories, and four experiments were performed. ConvNet#4 was not implemented on the images because the total filters of convolutional layers and pooling operations exceeded the input image dimensions. When the blurred appearance of X-ray images was considered, image sharpening using a Laplacian filter (sigma = 2.5)^21^ was applied to improve the visual appearance of the images and to test the ConvNets with different input data.

**Figure 1.**
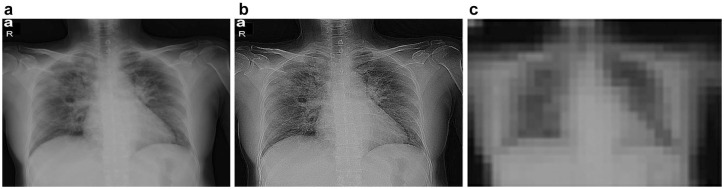
Pre-process of X-ray images. (a) Original chest X-ray image, (b) sharpened image using a Laplacian filter, and (c) average pixel per node (APPN)-applied image (10× enlarged).

The APPN approach, which is widely and efficiently used in image pre-processing for classification tasks,^4^ was applied to the X-ray images to obtain statistically resized images. APPN is based on dividing the image into segments with predetermined sizes, and taking the mean of the pixels within the corresponding segment. Thus, statistically reduced dimensions of images are obtained. In this research, 640×480 X-ray images were resized to 600×400, and then APPN with 20×20 segment sizes was applied. As a result, 30×20 X-ray images were produced. **Figure 1** presents the original, sharpened, and APPN-applied X-ray images.

In addition to these, original images were sent to ConvNets without any pre-processing. All experiments were performed on four different ConvNet architectures with two different image dimensions. **Table 2** shows the properties of ConvNet experiments.

#### Statistical Measurement Experiments

Each image hides basic statistical information that is useful for machine learning models. Consideration of the limited number of values instead of images decreases the computational time while achieving reasonable results. In this research, basic statistical information and the pre-processed characteristics were obtained from the images.

A threshold value was determined as half of the maximum pixel value within the image, and the number of pixels greater and smaller than this value were counted. Then, the image was divided into three segments vertically, and the center one was the widest so as not to divide the region of interest. The mean values of each segment were calculated separately. This process was performed to eliminate the corners and borders within the image. The mean values of Laplacian filter, sharpened image, and histogram equalization applied images were calculated separately to provide different information to the machine learning models for the same image at the same time. Besides these measurements, the minimum and maximum pixel values within the image, image entropy, standard deviation, variance, and the mode were calculated. **Table 3** shows the created statistical and fundamental properties of the images in detail. A feature vector with 14 attributes, described above, was created and fed to five machine learning classifiers: SVM, LR, nB, DT, and kNN.

**Table 3.** Description of Feature Vectors Created from X-Ray Images.

Attribute	Description
Lower	Total number of pixel values smaller than [max(p)/2]
Higher	Total number of pixel values greater than [max(p)/2]
LMean	Mean of the left segment of image
CMean	Mean of the center segment of image
RMean	Mean of the right segment of image
MeanLP	Mean of the Laplacian filter
MeanSh	Mean of the sharpened image
MeanHE	Mean of the histogram equalization applied image
Min	Minimum pixel value within the image
Max	Maximum pixel value within the image
Entropy	Entropy of the image
StdDev	Standard deviation of the image
Var	Variance of the image
Mode	Pixel value that is the most frequent within the image

#### Transfer Learning Experiments

The images that gave the best results with the ConvNet experiments and statistical measurement experiments, which were the unprocessed images, were compared with the pre-trained networks mentioned in the previous section.

VGG16^13^ is a CNN architecture that has 16 layers with weights and uses 3×3 filters. After convolutional layers, it has two fully connected layers, followed by a softmax for output. It has approximately 138 million parameters for the network. VGG19^13^ is similar to VGG16, but it has 19 layers with weights, and this provides approximately 143 million parameters for the network.

ResNet50^14^ has 50 residual layers, which aim to solve problems such as time consumption when the network becomes deeper. Its principle is based on skip connections between layers called identity function, and this increases the accuracy of the model and decreases the training time. It has more than 23 million trainable parameters.

Inception V3^15^ has 42 layers and 24 million parameters. It factorizes convolutions to reduce the number of parameters without decreasing the network efficiency. In addition, novel downsizing was proposed in Inception V3 to reduce the number of features.

MobileNet-V2^16^ has 53 layers and more than 3.4 million trainable parameters. It consists of residual connections and expansion, depthwise, and projection convolutions. The expansion convolutions convert the input tensor into a higher-channel tensor; depthwise convolutions apply filters to the converted tensors; and, finally, the projection convolutions project the higher channels to a smaller number of tensors.

DenseNet121^17^ connects each layer to every other layer in a feedforward fashion. The initial convolutional layer is followed by a fully connected layer, and the rest of the convolutional layers are followed by the pooling and a fully connected layer. It has 121 layers and more than 8 million trainable parameters.

Each X-ray image was sent to the considered networks with the minimum dimensions required. The pre-processing was performed on the considered models’ pre-processing steps to provide consequent images to the models. After training of each model with pre-trained weights, maximum pooling was applied, and features were sent to the fully connected layer (128). Similar to previous experiments, the eightfold cross-validation method was used for all experiments.

#### Model Evaluation Criteria

Models can be evaluated using different criteria, such as classification accuracy, sensitivity (true positive rate), specificity, and ROC AUC. Using only an accuracy or a sensitivity/specificity criterion is not enough, however, especially for imbalanced data; while higher scores can be obtained in one metric, lower scores can be produced by other metrics. Therefore, considering all the above-mentioned criteria, ROC AUC was used to evaluate the model performance for the statistical measurement, COVID-19/Normal, and COVID-19/Pneumonia experiments, which had two output classes (labels). ROC AUC is used to measure the performance of a model. In medical applications, the model with the higher ROC AUC score is more capable of distinguishing between patients with COVID-19 and without COVID-19.^22^ “Positive” and “negative” results are the responses of the outputs (classification predictions) obtained from the model. “True” and “false” are the actual data. The accuracy, sensitivity, and specificity are calculated as given in Equation (1), Equation (2), and Equation (3), respectively:

(1) Accuracy=(TP+TN)/(TP+TN+FP+FN)

(2) Sensitivity=TP/(TP+FN)

(3) Specificity=TN/(TN+FP)

where TP and TN denote the true-positive and true-negative values, respectively; and FP and FN represent false-positive and false-negative values, respectively.

Macro-averaged F1 score is a measure of model performance for multiclass (multilabel) problems that have more than two output classes, if the data are imbalanced, and the accuracy is not reliable.^23^ It considers the harmonic mean of recall and precision scores of all classes separately, and measures the capacity of the model for the correct detection of samples.

All experiments were performed by *k*-fold cross-validation,^24^ which is based on dividing all the data into a predefined number of folds, *k*, and using onefold for testing and the remaining for training. The training step is repeated *k* times until all folds are used for the test set. In this study, eightfold cross-validation was used for testing.^25^ Therefore, 12.5% and 87.5% of the data were used for testing and training, respectively. Four randomly selected images for both healthy and coronavirus-infected patients were assigned as the validation set. The number of images within the validation set was limited so as not to reduce the number of images in the infected class.

At the end of statistical measurement, COVID-19/Normal, and COVID-19/Pneumonia experiments, the mean accuracy, mean specificity, mean sensitivity, and the mean ROC AUC scores were calculated, and all the evaluations were performed on the mean scores. The mean ROC AUC scores were, however, used as the primary evaluation criteria. For COVID-19/Pneumonia/Normal experiments, the macro-averaged F1 score was used for the model evaluation. All experiments were performed on an Ubuntu 18.01.4 LTS 64-bit operating system, Intel Core i7-8700 CPU @3.20 GHz × 12, 32 GB RAM, NVidia GeForce RTX2060 GPU.

## Results

This section presents the results obtained from ConvNet experiments, statistical measurement experiments, and transfer learning experiments.

### Results of ConvNet Experiments

As mentioned above, 38 experiments were performed for the ConvNet experiments in three groups separately.

#### Results of COVID-19/Normal Experiments

In this group, a total of 1808 images (225 COVID-19 and 1583 Normal) were trained in each experiment without the data augmentation procedure, which artificially increases the training samples.

Sharpened images with different image sizes and by using different architecture produced consistent results for all experiments (Exp.1 through Exp.7). The highest mean accuracy of Experiments 1–7 was obtained in Exp.3 (98.34%). The highest mean sensitivity, highest mean specificity, and highest mean ROC AUC score, which is the primary indicator for an imbalanced dataset, however, were obtained in Exp.1 (91.05, 99.61, and 95.33%, respectively). Exp.2 and Experiments 3–7 could not achieve higher rates than Exp.1 and Exp.3 in all evaluation metrics.

In the APPN-applied experiments (Exp.8, Exp.9, and Exp.10), while the higher mean accuracy, higher mean sensitivity, and higher ROC AUC score were obtained in Exp.8 (98.23, 91.84, and 95.41%, respectively), the higher mean specificity was achieved in Exp.10 (99.29%). Exp.9, which was implemented using the deepest ConvNet architecture for APPN, produced the lowest results within these three experiments.

In Exp. 11 through Exp. 17, in which original images were used with different dimensions in different ConvNet architectures, consistent rates were obtained for mean accuracy and mean specificity. Changes in the rates of mean sensitivity and mean ROC AUC scores (between 3 and 6%, respectively) were, however, obtained using the different architectures. The highest mean accuracy and highest mean specificity were obtained in Exp.14 (99.11 and 99.78%), and these were the highest scores obtained in the ConvNet experiments for the COVID-19/Normal group. The highest mean sensitivity and highest mean ROC AUC scores for the COVID-19/Normal group were achieved in Exp.11 with 93.84 and 96.51%, respectively. **Table 4** shows the results obtained in the experiments for COVID-19/Normal classification.

**Table 4.** Results Obtained for COVID-19/Normal and COVID-19/Pneumonia Classification.

	COVID-19/Normal				COVID-19/Pneumonia			
Experiment	Mean Sensitivity (%)	Mean Specificity (%)	Mean Accuracy (%)	Mean ROC AUC (%)	Mean Sensitivity (%)	Mean Specificity (%)	Mean Accuracy (%)	Mean ROC AUC (%)
Exp.1	91.05	99.61	98.33	95.33	89.77	99.58	99.09	94.67
Exp.2	87.55	99.32	97.05	92.70	88.00	99.60	99.02	93.80
Exp.3	90.98	99.37	98.34	95.17	89.33	99.51	99.00	94.42
Exp.4	86.63	99.60	98.05	92.00	85.33	99.67	98.95	92.50
Exp.5	90.12	98.42	97.40	94.27	87.55	99.51	98.91	93.53
Exp.6	86.88	98.05	96.78	93.66	85.33	99.44	98.73	92.38
Exp.7	89.19	99.23	98.00	94.21	84.88	99.32	98.60	92.00
Exp.8	91.84	98.98	98.23	95.41	84.44	99.62	98.87	92.03
Exp.9	87.33	98.97	97.13	93.69	85.33	99.37	98.67	92.35
Exp.10	88.67	99.29	97.95	93.98	88.00	99.51	98.93	93.75
Exp.11	93.84	99.18	98.50	***96.51***	92.88	99.79	99.44	***96.33***
Exp.12	88.37	99.57	98.91	93.89	87.11	99.62	99.00	93.36
Exp.13	87.88	98.98	97.73	93.43	87.11	99.62	99.00	93.36
Exp.14	89.12	99.78	99.11	94.57	85.77	99.18	98.51	92.48
Exp.15	90.10	99.50	98.34	94.80	90.22	99.67	99.20	94.94
Exp.16	84.11	98.80	97.64	91.01	86.22	99.60	98.93	92.91
Exp.17	87.71	99.11	97.73	93.41	86.22	99.48	98.82	92.85

COVID-19: Coronavirus disease 2019; ROC AUC: receiver operating characteristics–area under the curve.

#### Results of COVID-19/Pneumonia Experiments

In the second group of ConvNet experiments, a total of 4517 images (225 COVID-19 and 4292 Pneumonia) were trained in each experiment; as with the COVID-19/Normal experiments, the data augmentation procedure was not applied. Even though the number of training images was increased, and the second training set (Pneumonia set) is a challenging dataset for detecting COVID-19, similar results to those in the COVID-19/Normal experiments were obtained.

In sharpening applied experiments (Experiments 1–7), the mean ROC AUC scores fluctuated up to 2.4%, and the highest mean ROC AUC score (94.64%), highest mean sensitivity (89.77%), and highest mean accuracy (99.09%) were obtained in Exp.1 that was implemented using ConvNet#2. But the highest mean specificity achieved was 99.60% in Exp.4. In APPN-applied experiments (Experiments 8–10), similar results were obtained; however, the lightest architecture achieved the highest mean ROC AUC score.

When the images fed ConvNets directly (Experiments 11–17), we observed that the increment of the convolutional layer number of ConvNets reduces the scores obtained by the neural network up to 4%, similar to COVID-19/Normal results. The highest mean accuracy, mean sensitivity, mean specificity, and mean ROC AUC scores were obtained in Exp.11: 99.44, 92.88, 99.79, and 96.33%, respectively. **Table 4** shows the results obtained in the experiments for the COVID-19/Pneumonia classification.

#### Results of COVID-19/Pneumonia/Normal Experiments

In the last group of ConvNet experiments, a total of 6100 images (225 COVID-19, 4292 Pneumonia, and 1583 Normal) were trained in each experiment for three output classes as COVID-19, Normal, and Pneumonia. Because superior results were obtained without image pre-processing in COVID-19/Normal and COVID-19/Pneumonia experiments, the experiments in this group were performed using only the unprocessed images with 160×120 dimensions with four considered ConvNet architectures.

ConvNet#1 could not achieve the highest scores in any metrics in terms of recall and precision for each class, and it produced 92.70% for a macro-averaged F1 score in COVID-19/Pneumonia/Normal experiments. ConvNet#1, which was the lightest structure and produced the optimal results in two-class experiments, could not produce the highest results in three-class experiments. The results obtained by ConvNet#1 were similar to ConvNet#3 results, and the macro-averaged F1 score was 92.84%.

ConvNet#3 achieved the highest results obtained in this group in terms of precision, recall, and F1 score for all classes. The macro-averaged F1 score was 94.10%. ConvNet#4, with the deepest structure, produced similar results to ConvNet#2 but could not outperform it. It achieved a macro-averaged F1 score of 94.04%. **Table 5** presents the results obtained in COVID-19/Pneumonia/Normal experiments.

**Table 5.** Results Obtained for COVID-19/Pneumonia/Normal Classification.

	Mean Precision (%)			Mean Recall (%)				
Model	Corona	Normal	Pneumonia	Corona	Normal	Pneumonia	Mean Accuracy (%)	Macro-Averaged F1 Score (%)
DenseNet121	98.87	90.90	88.52	95.66	97.20	92.03	95.99	93.85
Inception V3	97.76	90.99	86.54	95.99	96.54	91.16	94.90	93.14
ConvNet#1	96.20	93.72	92.98	97.45	86.22	90.63	95.26	92.84
ConvNet#2	96.77	95.27	93.05	97.41	90.04	92.12	95.75	**94.10**
ConvNet#3	96.26	93.15	92.26	96.98	86.79	90.88	95.04	92.70
ConvNet#4	97.51	91.42	92.32	96.90	92.69	93.49	95.88	94.04

ConvNet: Convolutional neural network; COVID-19: coronavirus disease 2019.

### Results of Statistical Measurement Experiments

Five experiments were performed for COVID-19/Normal classification by considering 14 features obtained from the images and using five machine learning classifiers: SVM, LR, nB, DT, and kNN. Inconsistent results were obtained for kNN and nB. kNN achieved the highest mean specificity rate (99.55%), but it also produced the lowest mean sensitivity and lowest mean ROC AUC score (63.10 and 81.33%, respectively). Similarly, nB produced the highest mean sensitivity rate and mean ROC AUC score (82.95 and 92.75%, respectively), but it produced the lowest mean accuracy and mean specificity rates (93.97 and 94.05%, respectively). SVM achieved the highest mean accuracy result (96.57%). None of these models, however, was capable of outperforming the ConvNet for any of the evaluation metrics using the obtained statistical data. **Table 6** presents the results obtained in statistical measurement experiments.

**Table 6.** Results Obtained in Statistical Measurement Experiments.

	COVID-19/Normal				COVID-19/Pneumonia			
Experiment	Mean Sensitivity (%)	Mean Specificity (%)	Mean Accuracy (%)	Mean ROC AUC (%)	Mean Sensitivity (%)	Mean Specificity (%)	Mean Accuracy (%)	Mean ROC AUC (%)
SVM	81.30	98.80	96.57	90.05	75.55	97.85	96.74	86.70
Logistic Reg.	68.36	98.12	94.41	83.24	66.66	96.45	94.97	81.56
Decision Tree	75.91	96.53	93.97	87.10	69.77	96.50	95.17	83.14
Naive Bayes	82.95	94.05	93.97	**92.75**	80.00	97.85	96.96	**88.92**
kNN	63.10	99.55	95.02	81.33	64.44	96.22	94.64	80.33

COVID-19: Coronavirus disease 2019; kNN: k-nearest neighbor; ROC AUC: receiver operating characteristics–area under the curve (AUC); SVM: support vector machine.

The same machine learning classifiers and features were considered for the classification of COVID-19/Pneumonia. Similar results were obtained in the experiments, and nB produced the highest mean ROC AUC, mean sensitivity, and mean accuracy scores (88.92, 80.00, and 96.96%, respectively) for statistical measurement experiments of COVID-19/Pneumonia classification. The highest mean specificity was obtained by nB and SVM (97.85% each). The lowest scores of statistical measurements for COVID-19/Pneumonia classification were obtained by LR. Even though similar results were obtained in COVID-19/Normal and COVID-19/Pneumonia experiments, the decrement in the classification levels was observed for all machine learning algorithms. This might be caused by both image classes having disease and the increment of the number of training images.

### Transfer Learning Experiments

Comparisons were performed for all groups of experiments. Pre-processing methods were not applied to the images because the original images achieved the highest results in ConvNet experiments. Similar to ConvNet experiments, transfer learning experiments were also performed in three groups as COVID-19/Normal, COVID-19/Pneumonia, and COVID-19/Pneumonia/Normal. The two models that would produce superior results in the COVID-19/Normal and COVID-19/Pneumonia groups were considered in COVID-19/Pneumonia/Normal experiments.

In the COVID-19/Normal group, VGG19 and MobileNet-V2 produced the worst results. They were only able to learn one class and could not classify COVID-19 X-ray images. ResNet-50 and VGG16 produced comparatively better results than VGG19 and MobileNet-V2. The mean ROC AUC scores of ResNet-50 and VGG16 were calculated as 65.78 and 72.64%, respectively. Inception-V3 produced higher results than other pre-trained networks; however, the highest mean ROC AUC score in transfer learning experiments was obtained by DenseNet121 (96.48%). **Table 7** presents the results obtained using transfer learning for the COVID-19/Normal group.

**Table 7.** Results Obtained in Transfer Learning Experiments for COVID-19/Normal and COVID-19/Pneumonia Classification.

	COVID-19/Normal				COVID-19/Pneumonia			
Exp.	Mean Sensitivity (%)	Mean Specificity (%)	Mean Accuracy (%)	Mean ROC AUC (%)	Mean Sensitivity (%)	Mean Specificity (%)	Mean Accuracy (%)	Mean ROC AUC (%)
VGG16	46.04	99.24	92.64	72.64	77.33	99.65	98.53	88.49
VGG19	08.03	100.0	88.55	54.01	70.66	99.48	98.05	85.07
InceptionV3	90.14	99.17	98.17	94.66	89.77	99.65	99.15	94.71
MobileNet-V2	08.40	100.0	87.61	54.20	68.88	99.39	97.87	84.14
ResNet50	31.57	100.0	91.15	65.78	59.55	100.0	97.98	79.77
DenseNet121	93.92	99.04	98.39	**96.48**	92.44	99.46	99.11	**95.95**

COVID-19: Coronavirus disease 2019; ROC AUC: receiver operating characteristics–area under the curve (AUC).

In the COVID-19/Pneumonia group, similar results were obtained. Even though the VGG19, MobileNet-V2, and ResNet50 increased their scores, they were not able to reach the scores of DenseNet121 and Inception V3. The highest mean ROC AUC score of COVID-19/Pneumonia classification in the transfer learning experiment was achieved by DenseNet121 (95.95%), and it was followed by Inception V3 (94.71%). **Table 7** presents the results obtained using transfer learning for the COVID-19/Pneumonia group.

After considering the results obtained in the first two groups, we implemented DenseNet121 and Inception V3 for the classification of COVID-19/Pneumonia/Normal. Even though fluctuating results were observed for precision and recall scores for the COVID-19, Pneumonia, and Normal classes, DenseNet121 outperformed Inception V3 in transfer learning experiments by obtaining a macro-averaged F1 score of 93.85%, while Inception V3 achieved 93.14%. **Table 5** shows the results obtained in COVID-19/Pneumonia/Normal experiments with the results obtained in ConvNet experiments of the same group.

### Comparisons of Experiments

In COVID-19/Normal classification, the highest mean specificity (when the 100.0% scores of pre-trained networks are not considered because of not learning another class) and the highest mean accuracy results were obtained in Exp.14 (99.78 and 99.11%, respectively), which consisted of the deepest architecture in ConvNet experiments (**Table 4**). This failed, however, to produce higher results in terms of mean sensitivity, and this reduced the performance of the considered ConvNet in the primary performance indicator for both classes, mean ROC AUC score. The highest mean sensitivity was achieved by DenseNet121 (93.92%) (**Table 7**), but other obtained scores were not high enough to outperform other models in other metrics. DenseNet121’s mean ROC AUC score was 96.48%. Even though ConvNet#1 could not produce the optimal results in sensitivity, specificity, and accuracy results, its stability produced consistent results, and the highest mean ROC AUC score was achieved by ConvNet#1 with 96.51% (**Table 4**). Machine learning classifiers could not produce satisfactory results using the extracted statistical information to classify COVID-19 in this experimental group.

**Figure 2.**
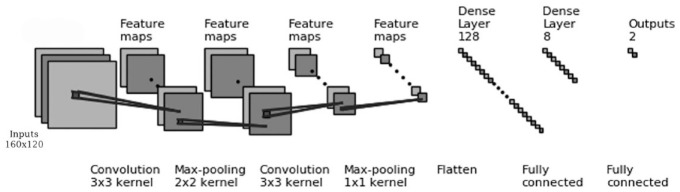
Convolutional neural network 1 (ConvNet#1) architecture with two convolutional and two fully connected layers.

**Figure 3.**
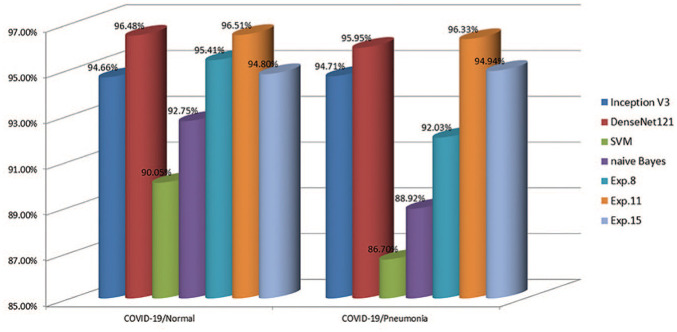
Highest ROC AUC scores obtained in the COVID-19/Normal and COVID-19/Pneumonia experiments. COVID-19: Coronavirus disease 2019; ROC AUC: receiver operating characteristics–area under the curve.

In COVID-19/Pneumonia classification, similarly to the previous experiments, the highest mean ROC AUC score was obtained in Exp.11 (96.33%) with ConvNet#1 (**Table 4**), followed by DenseNet121 (95.95%) (**Table 7**). Besides, the highest mean sensitivity and mean accuracy results were also obtained in Exp.11 (92.88 and 99.44%, respectively). The highest mean specificity was achieved in transfer learning experiments by ResNet50 (100%); however, the other results reduced the success of the model to classify two-class experiments correctly at the same time. Similarly to the previous experiments, machine learning experiments could not produce similar results to those of ConvNet experiments and transfer learning experiments. **Table 8** shows the total TP, TN, FP, and FN results obtained for Exp.11 and DenseNet121 for all folds in COVID-19/Normal and COVID-19/Pneumonia classification. **Figure 2** demonstrates the architecture of ConvNet#1, which obtained the highest classification results, and **Figure 3** shows some of the highest ROC AUC scores obtained in ConvNet, statistical measurement, and transfer learning experiments.

**Table 8.** TP, FP, TN, and FN results for Exp.11 and Densenet121 for all test folds.

COVID-19/Normal				
Experiment	TP	FP	TN	FN
Exp.11	**211**	15	1568	14
Densenet121	209	**13**	**1572**	14
COVID-19/Pneumonia				
Experiment	TP	FP	TN	FN
Exp.11	**209**	**9**	**4283**	**16**
Densenet121	208	23	4269	17

COVID-19: Coronavirus disease 2019; FN: false negative; FP: false positive; TN: true negative; TP: true positive.

**Figure 4.**
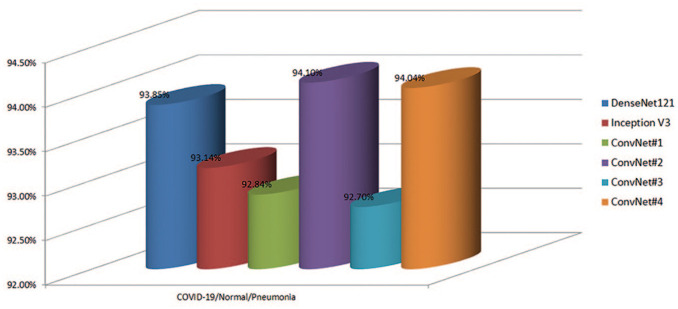
Macro-averaged F1 scores of the COVID-19/Normal/Pneumonia experiments. COVID-19: Coronavirus disease 2019.

For three-class experiments (COVID-19/Pneumonia/Normal), the macro-averaged F1 scores were between 92.70 and 94.10% (**Table 5**). DenseNet121, however, achieved higher results than ConvNet#1, ConvNet#3, ConvNet#4, and Inception V3. But the optimal results were obtained by ConvNet#2, which had a macro-averaged F1 score of 94.10%, followed by DenseNet121 with 93.85%, as shown in **Table 5**. **Figure 4** shows the macro-averaged F1 scores obtained in COVID-19/Normal/Pneumonia experiments.

## Discussion

The performed experiments should be analyzed separately to evaluate the performance of the applied techniques and considered models. As mentioned above, the final evaluation process was performed by the eightfold cross-validation method and ROC AUC score because of the imbalanced database.

In two-class experiments, a variety of image pre-processing methods were applied with different image sizes and four ConvNet architectures to provide the highest detection accuracy of COVID-19 in chest X-ray images.

In COVID-19/Normal classification experiments, it was relatively easier to classify COVID-19 because the normal X-ray images do not contain any abnormalities. The performed experiments showed that the considered image pre-processing steps produced similar results to ConvNets fed with original images; however, none of these considered techniques were able to increase the performance of ConvNets in terms of mean ROC AUC score. The maximum mean ROC AUC score using an image pre-processing technique was 95.41%, which was obtained in Exp.8 with ConvNet#1 and APPN. The use of the images with reduced dimensions caused the mean ROC AUC scores of the experiments to decrease by approximately 5.5% (max. 96.51% and min. 91.01%) compared to the experiments with higher dimensions. A possible solution is feeding the ConvNet with images with increased dimensions.

Four architectures were also considered for all experiments to evaluate the model performance with different numbers of layers. Experimental results showed that the use of more convolutional and fully connected layers could not improve the model performance for the image database considered, because the differences between the mean ROC AUC scores of the ConvNet with minimized layers and the ConvNet with more layers were more than 1.7–5%, depending on the pre-processing technique. The minimum mean ROC AUC score of ConvNet with more layers in APPN-applied images was 93.69%, while ConvNet#1 achieved 95.41%. The number of images used in the experiments has a direct effect on the number of layers and the architecture of the ConvNet, but the obtained results suggest that the use of minimized layer numbers can enhance detection of COVID-19 within the normal images. The highest result was obtained by using two convolutional layers and two dense layers with 160×120 image dimensions.

Then, statistical measurements and COVID-19 detection using several machine learning models were considered. The determination of the specific statistical measurements to be used is vital for this kind of classification approach; however, there are basic measurements that can be obtained from the images. In addition to the above-mentioned statistical measurements, the image pre-processing techniques were applied, and additional measurements were obtained from the images to make the knowledge for the machine learning models as similar as possible to that for the ConvNets. The machine learning models, however, could not achieve mean ROC AUC scores as high as those of the ConvNets, and there was a 4% difference between the highest mean ROC AUC score in ConvNet experiments and nB, which produced the highest result in statistical measurement experiments.

The use of transfer learning with the state-of-the-art pre-trained ConvNets was also considered in COVID-19/Normal classification experiments. Six pre-trained networks were considered, and the results showed that two of them, InceptionV3 and Densenet121, were able to correctly detect the X-ray images. Densenet121 produced similar results to the highest results obtained in Exp.11; however, it could not outperform Exp.11 in terms of mean specificity, mean accuracy, and mean ROC AUC scores.

The other classification type in this study was the detection of COVID-19 within the pneumonia images (COVID-19/Pneumonia). The same experiments were performed as with COVID-19/Normal experiments, and similar results were obtained. The lightest ConvNet outperformed the other considered ConvNet structures and pre-trained models, even though the number of training samples increased because of the number of images in the dataset. Similarly, machine learning classifiers were not able to produce higher results than ConvNets obtained, but general reduction was observed in the classification performance of machine learning models. This was caused by the complexity of images, the difficulty of differentiating COVID-19 from pneumonia images, and the increased number of training samples. It should be noted, however, that additional measured characteristics of images or significant statistical measurements, such as contrast level, brightness level, kurtosis, and so on, may help to improve the scores obtained by machine learning models.

In three-class experiments (COVID-19/Normal/Pneumonia), the increment of the class number and the training samples caused ConvNet#1 to not produce optimal results. Even the deepest structure (ConvNet#4) could not achieve superior results; it was observed that the deeper structure was more effective than ConvNet#1 at detecting COVID-19 between pneumonia and normal images.

Although the success of the recognition ability of the models strongly depends on the image or dataset characteristics, we can conclude that the use of lighter ConvNets for a smaller number of output classes for a limited number of images performs better convergence. The increment of the number of output classes and training samples, however, requires a deeper structure for effective learning. It should also be noted that the characteristics of the images have a direct effect on convergence; therefore, different architectures should be analyzed for each application to improve the recognition capacity of the model.

Pre-trained networks have very deep architectures, they have been trained by using millions of different kinds of images, and the saved final weights are intended to be transferred to similar or different applications. Recent research,^26–28^ however, aimed to develop light ConvNets to reduce the computational cost of pre-trained networks; and, as mentioned above, networks with less deep architectures become preferable for classification problems, even with a huge number of images and a high number of output classes. The obtained results also demonstrate that architectures may begin to deepen more in connection with the increased number of images and output classes. For this reason, some pre-trained neural networks have been found to have difficulties in learning one class successfully while learning another class with high accuracy. Similar results were obtained in Apostolopoulos and Mpesiana.^2^

COVID-19 data used in this study have been collected by pulling images from publications and websites. Therefore, they have come from different institutions and different scanners. X-ray imaging parameters might be different for some of the scans, which might result in different image quality, and this is common when multisite studies are mixed, or one database has multiple characteristic flaws like different imaging protocols. Therefore, pre-processing of the data to make the radiographic images more similar and uniform is important in terms of providing more efficient analysis and consistency. This is a complex procedure, however, including co-registration, standardization, and so on to obtain the same image size and pixel size along the same spatial orientation and to make the images’ resolution uniform and isotropic. We believe that, as more pre-processed datasets on COVID-19 become publicly available, more accurate studies will be conducted. Nevertheless, the current limited dataset has led researchers around the globe to develop methods to aid in facilitating the diagnosis of COVID-19. Although this study shows that CNNs can be used for automated detection of COVID-19 and for distinguishing it from pneumonia, we believe applying artificial neural networks to COVID-19 detection more accurately requires clinical trials.

Another limitation of this study is the small sample size of COVID-19 images, which restricts the appropriate cohort selection and might result in a biased conclusion. At the time of writing, there is no other reliable publicly available dataset. To have a more accurate and robust model, a larger COVID-19 dataset is needed. Furthermore, because of the use of a relatively small number of COVID-19 images, clinical information about the patients, such as risk factors and medical history, is not available at this time.

## Conclusions

Detection of COVID-19 from chest X-ray images is of vital importance for both doctors and patients to decrease the diagnostic time and reduce financial costs. Artificial intelligence and deep learning are capable of recognizing images for the tasks taught. In this study, several experiments were performed for the high-accuracy detection of COVID-19 in chest X-ray images using ConvNets. Various groups—COVID-19/Normal, COVID-19/Pneumonia, and COVID-19/Pneumonia/Normal—were considered for the classification. Different image dimensions, different network architectures, state-of-the-art pre-trained networks, and machine learning models were implemented and evaluated using images and statistical data. When the number of images in the database and the detection time of COVID-19 (average testing time = 0.03 s/image) are considered using ConvNets, it can be suggested that the considered architectures reduce the computational cost with high performance. The results showed that the convolutional neural network with minimized convolutional and fully connected layers is capable of detecting COVID-19 images within the two-class, COVID-19/Normal and COVID-19/Pneumonia classifications, with mean ROC AUC scores of 96.51 and 96.33%, respectively. In addition, the second proposed architecture, which had the second-lightest architecture, is capable of detecting COVID-19 in three-class, COVID-19/Pneumonia/Normal images, with a macro-averaged F1 score of 94.10%. Therefore, the use of AI-based automated high-accuracy technologies may provide valuable assistance to doctors in diagnosing COVID-19.

Further studies, based on the results obtained in this study, would provide more information about the use of CNN architectures with COVID-19 chest X-ray images and improve on the results of this study.
